# Green-Synthesized Silver Nanoparticles Induced Apoptotic Cell Death in MCF-7 Breast Cancer Cells by Generating Reactive Oxygen Species and Activating Caspase 3 and 9 Enzyme Activities

**DOI:** 10.1155/2020/1215395

**Published:** 2020-10-05

**Authors:** Ikram Ullah, Ali Talha Khalil, Muhammad Ali, Javed Iqbal, Waqar Ali, Saud Alarifi, Zabta Khan Shinwari

**Affiliations:** ^1^Department of Biotechnology, Quaid-i-Azam University, Islamabad 45320, Pakistan; ^2^Qarshi Herb Research Centre, Qarshi Industries Pvt. Ltd., Hattar-Haripur, Pakistan; ^3^Department of Pathology, Medical Technology Institution, Lady Reading Hospital, Peshawar, Pakistan; ^4^Department of Plant Sciences, Quaid-i-Azam University, Islamabad 45320, Pakistan; ^5^Department of Biotechnology, University of Malakand, Chakdara, Lower Dir, Pakistan; ^6^Department of Zoology, College of Science, King Saud University, P.O. Box 2455, Riyadh 11451, Saudi Arabia; ^7^Pakistan Academy of Sciences, Islamabad, Pakistan

## Abstract

Silver nanoparticles are among the most significant diagnostic and therapeutic agents in the field of nanomedicines. In the current study, the green chemistry approach was made to optimize a cost-effective synthesis protocol for silver nanoparticles from the aqueous extract of the important anticancer plant *Fagonia indica*. We investigated the anticancer potential and possible involvement of AgNPs in apoptosis. The biosynthesized AgNPs are stable (zeta potential, -16.3 mV) and spherical with a crystal size range from 10 to 60 nm. The MTT cell viability assay shows concentration-dependent inhibition of the growth of Michigan Cancer Foundation-7 (MCF-7) cells (IC_50_, 12.35 *μ*g/mL). In addition, the fluorescent microscopic analysis shows activation of caspases 3 and 9 by AgNPs that cause morphological changes (AO/EB assay) in the cell membrane and cause nuclear condensation (DAPI assay) that eventually lead to apoptotic cell death (Annexin V/PI assay). It was also observed that AgNPs generate reactive oxygen species (ROS) that modulate oxidative stress in MCF-7 cells. This is the first study that reports the synthesis of a silver nanoparticle mediated by *Fagonia indica* extract and evaluation of the cellular and molecular mechanism of apoptosis.

## 1. Introduction

Cancer of the breast is the most important cause of mortalities in women all over the globe. Various chemotherapeutic treatments can be applied for its treatment. However, they are costly and accompanied by frightening side effects. In addition, cells in breast cancer are becoming resistant to the various available chemotherapies [1]. Therefore, it is mandatory to search for alternative treatment options that are efficient, cost-effective, and biocompatible. Recently, metal and metal oxide nanoparticles have developed as a stimulating area of research because of their widespread applications [2–8]. Nanobiotechnology has noteworthy applications in biomedical sciences as a field aiding therapeutics and diagnostics [9–11]. Recently, AgNPs have been comprehensively researched because of their interesting physical, biochemical, and therapeutic properties [12, 13].

Numerous chemical and physical methods are adopted for the synthesis of AgNPs [14]. Although these methods are effective, they come with some shortcomings. The chemical and physical methods require high energy resources and generate toxic by-products and hazardous wastes [5, 15]. Recent researches indicate the presence of noxious chemicals with the chemically synthesized nanoparticles making them relatively unfavourable for biomedical applications [16]. To overcome the issue of energy balance and toxic by-products, complete green methods are used for the synthesis of AgNPs. Therefore, a paradigm shift is observed towards the biological synthesis of AgNPs. Many biological assets like plants, microbes, algae, and yeasts are used to synthesize nanoparticles [17]. However, due to their ample availability, low cost and a rich source of bioreducing agents, plants, and plant products are the most frequently used approach for the synthesis of nanoparticles [18, 19]. Biosynthesis is beneficial over other methods because of its low cost, rapidity, single step synthesis, high yield, and biocompatibility [20]. Furthermore, the size can be controlled easily by adjusting the salt concentrations, pH, and temperature.

The interface of medicinal plants, nanoparticles, and cancer is an exciting horizon for the search of alternative and cheap chemotherapeutics. Herein, we optimized a complete green protocol for the phytosynthesis of AgNPs via aqueous extracts of the medicinally important plant *Fagonia indica*. Indigenous knowledge reveals potential anticancer properties of *Fagonia indica*. It is used in making herbal tea. Recent research also indicated the significant anticancer potential of *Fagonia* against MCF-7 cells [21]. Hitherto, the biological synthesis of AgNPs has been reported by researchers [13, 22, 23]; however, to date, no reports are available to study the anticancer mechanism of the phytosynthesized AgNPs mediated by the aqueous extract of *Fagonia indica*. This novel study describes the biosynthesis of AgNPs and investigates their cellular and molecular mechanisms of apoptosis caused by the *Fagonia* extract-mediated AgNPs. Earlier studies show various biological properties of biogenic AgNPs, such as antimicrobial [24–28], anticancerous [29–31], antiangiogenic, antiparasitic [32, 33], cytotoxic [34–37], and antitumor [38, 39]. AgNPs have differential effects in the killing of cancer cells. Sanpui and his coworkers demonstrated that AgNPs induced apoptosis by affecting the membrane integrity and normal cellular functions [40]. Vasanth and his colleagues reported apoptosis in human cervical cells by treatment with AgNPs [29].

Chemotherapy and combinational chemotherapy are still the common methods for the treatment of breast cancer [41]. However, due to their potential disadvantages, it is imperative to look for alternative and effective treatments. The current study was intended to synthesize silver nanoparticles by a simple and ecofriendly process using medicinal flora. Furthermore, the phytosynthesized AgNPs were studied for their anticancer activities in MCF-7 breast cancer cells while their anticancer mechanism is unveiled using different mechanistic assays.

## 2. Materials and Methods

### 2.1. Optimization of AgNP Biosynthesis

The stock solution of *Fagonia indica* aqueous extract (5 mg/mL) and AgNO_3_ (1 M) was prepared in distilled water. AgNO_3_ (extra pure, Merck) was diluted into 1, 2, 4, and 8 mM solutions. The extract and AgNO_3_ solution were mixed in the ratio of 1 : 2, 1 : 4, 1 : 8, and 1 : 10. The reaction mixtures were kept at room temperature and at 50, 60, 70, 80, and 90°C in the dark. The time-dependent data were taken at an interval of 10, 20, 40, 60, 90, and 120 min and 3, 4, and 5 hrs. The solution was stirred for 4 hours, and a color change was observed. For characterization, the mixture was centrifuged for 20 min at 13,000 rpm to get the pellet. The pellet was washed three times to remove any unbound plant compounds with distilled water by repeated centrifugation. Finally, the pellet was freeze-dried and lyophilized. The powder AgNPs obtained were further processed for characterization.

### 2.2. Characterization of AgNPs

UV-vis (JASCO, V-530) with a resolution of 1 nm in the range of 300 to 600 nm was used to observe the reduction of Ag^+^ ions in a colloidal solution. The zeta potential and hydrodynamic size were calculated using a Zetasizer (Malvern Instruments Ltd., U.K.). The crystalline nature and size of the nanoparticles were confirmed through X-ray diffraction analysis (X-ray diffractometer, Bruker D8 Advance) equipped with 40 kV/30 Ma X-ray, 2*θ*/*θ* scanning mode, CuK*α* radiation (*λ* = 1.5418 Å) and a fixed monochromator in the range of 20-80 degrees. The Scherrer approximation (*D* = *kλ*/*β*1/2 cos *θ*) was used to calculate the average size of the nanoparticles. FT-IR was carried out at Shimadzu (Shimadzu Corporation) to investigate the type of functional groups involved in the reduction and capping of nanoscale silver. The samples for electron microscopy were gold coated (JEOL, Model No. JFC-1600), and images were obtained by scanning electron microscope (ZEISS EVO-MA 10, Oberkochen, Germany).

### 2.3. Cell Culture

Dulbecco's modified Eagle's medium (DMEM) *pH* = 7.2 to which 10% FBS was supplemented was used to culture breast cancer MCF-7 cells. The media were also added with gentamicin (100 U/mL) to prevent any cross contamination. A humidified incubator with 5% CO_2_ was used to incubate cells. The cell population (80−90%) was harvested using trypsin then washed in PBS and used for further experiments.

### 2.4. Cell Viability Assays

The previously described protocol of MTT cytotoxicity assessment with slight modifications was used to investigate the viability of cells [42]. Cancer cells were grown to a density of 2 × 10^4^ cells/well for 24 hours and then exposed to different test concentrations of AgNPs for 24 hours. After, PBS with added 5.0 mg/mL MTT was introduced at the rate of 10 *μ*L into each well and incubated for another 4 hours. The introduction of MTT led to the formation of formazan crystals inside the live cells. DMSO (100 *μ*L) was introduced for dissolving the formazan crystals, and the readings were taken at 570 nm using a microplate ELISA reader (BioTek).

### 2.5. Morphological Study with Fluorescence Microscopy

AgNP-treated MCF-7 cells were investigated by AO/EB fluorescence staining techniques for determination of apoptosis [43]. Briefly, six-well plates were used to culture MCF-7 cells to a density of 1 × 10^5^ cells per well for 24 hours. Cells were then exposed to the already calculated inhibitory concentration (IC50_0_) for 24 hrs. Unexposed cells to AgNPs were taken as the control. A mixture of the AO/EB dyes (20 *μ*L) was prepared by mixing the two dyes prepared initially at 100 *μ*g/mL in PBS each. After staining the treated and control samples, these cells were monitored and imaged by fluorescence microscope (Olympus) with excitation (488 nm) and emission (520 nm).

### 2.6. Observation of Chromatin Changes

4,6-Diamidino-2-phenylindole (DAPI) staining assay was used to further examine the alterations of chromatin which is an integral part of the process of apoptosis [44]. The fluorescent dye DAPI was intended for the staining of nuclear DNA in cells which have undergone the process of apoptosis. Briefly, cells (MCF-7) were grown to the density of 1 × 10^6^ cells/well and introduced to the 24-well plates in the log phase followed by 24 h incubation. Afterwards, the IC_50_ concentrations of photosynthesized AgNPs were applied, and the culture was kept for up to 24 hours. After the treatment, 1x PBS was used to wash the cells which were fixed with 50 *μ*L of water and methanol mixed together in 1 : 1. 100 *μ*L of the 1 *μ*g/mL of the DAPI dye was used for staining followed by incubation at 37°C for 30 minutes in the dark. 20 *μ*L of PBS : glycerin (1 : 1) was introduced to remove the excess dye. Changes in chromatin were observed under inverted fluorescence microscope (40x). Apoptotic cells were expressed in percentage calculated as

(1) $$\begin{array}{c} \%\text{apoptotic cells}=\left(\frac{\text{amount of apoptotic nuclei}}{\text{amount of all cells}}\right)\times 100 \end{array}$$

### 2.7. Cell Apoptosis Assay

The quantification of the extent of apoptosis was performed by using Annexin V-FITC/PI double staining assay [45]. Only the IC_50_ concentration of AgNPs was considered for the treatment. Briefly, washing was carried out with PBS for the collected cells at least two times, and then staining was carried out with PI and Annexin V-FITC. Flow cytometry (Millipore Corporation, Billerica, MA, USA) was performed. Number of live cells, necrotic cells, late apoptotic cells, and early apoptotic cells were distinguished by direct counting of the cells.

### 2.8. Quantification of Caspase 3 and 9 Activities

Caspase 3 and 9 assay kits (Caspase-Glo® 3 and 9 reagents, Promega) were used to quantify caspase activities. Briefly, 50,000 MCF-7 cells/well were seeded in a 96-well plate. The cells were incubated in a 5% CO_2_ humidified incubator at 37°C for 24 hours. The 96-well plates containing AgNP-treated and AgNP-untreated control cells were then allowed to equilibrate at room temperature. 100 *μ*L of Caspase-Glo® 3 or 9 reagent was added to each well of a 96-well plate (test well and control) containing 100 *μ*L of culture medium. The plate was covered and the content mixed for 30 seconds at 500 rpm. The optical density was measured (ELISA reader, BioTek) at 405 nm after incubation of the plate at room temperature for 30 min.

### 2.9. ROS Assay in MCF-7 Cells

Dichlorofluorescein diacetate (DCFDA) probes were used to investigate the intracellular ROS production [46]. Briefly, the MCF-7 cells were seeded in 12-well plates for 24 hours and then treated with the IC_50_ concentration of AgNPs for 24 h. Trypsin EDTA was used to detach the cells. The cells were washed with PBS and resuspended in 200 *μ*L PBS containing a 10 mM DCFH-DA fluorescent probe. The reaction mixture was incubated for 30 minutes at 37°C. The extent of ROS generated was measured through a fluorescent spectrophotometer.

### 2.10. Data Analysis

Cytotoxicity of the nanoparticles was expressed as the concentration (*μ*g/mL) inhibiting the growth of 50% cells (IC_50_). Data was analyzed through MS Excel 2019, and IC_50_ was estimated through TableCurve 2D software. The graphs were prepared with OriginPro 8.1 and GraphPad.

## 3. Results and Discussions

### 3.1. Biosynthesis

**Figure 1:**
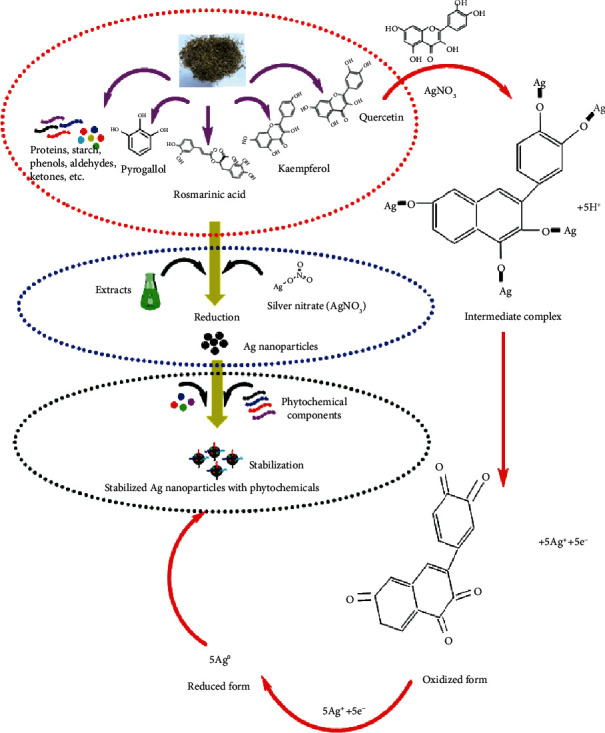
Proposed mechanism of silver ion reduction by plant metabolite into silver nanoparticles.

Biosynthesis of AgNPs using the green route has been optimized using the extract of *Fagonia indica*. Biological synthesis is considered the most adequate method compared to the physical and chemical means. Hitherto, while being effective, these physical and chemical synthesis methods are accompanied by certain disadvantages like cost, energy demands, and generation of toxic hazardous waste streams [15, 47]. Furthermore, in some reports, it was indicated that some toxic chemicals could remain adhered with the nanoparticles synthesized from chemical means which could not be used in biomedical applications [16, 48]. Therefore, biomodulated synthesis of AgNPs is preferred. *Fagonia indica* is a very important medicinal plant, and its therapeutic potential is well documented. Recent reports suggested the significant anticancer potential for the *Fagonia* species under in vitro conditions [49]. The medicinal potential of *Fagonia* is attributed to the novel phenolic and flavonoid chemical components, and these phytochemicals play the role of chelation and stabilize the nanoparticles in their biosynthesis. Although the biogenic synthesis of Ag nanoparticles has been reported successfully via plants, biosynthesis using medicinal pants with anticancer potential is rare. A mechanism proposed for the biosynthesis has been suggested in Figure 1.

### 3.2. UV-Vis Spectrophotometry

**Figure 2:**
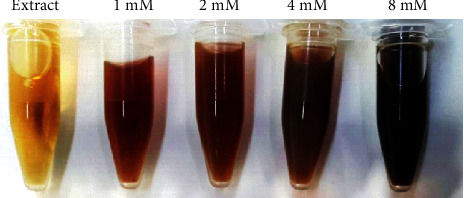
Variation in color intensity of green silver nanoparticles mediated at different AgNO_3_ concentrations by leaf extracts combined at the ratio of 1 to 10 (extract : AgNO_3_).

Biomodulated synthesis of AgNPs was optimized using different parameters. These parameters include optimization by (a) precursor concentration, (b) extract concentration, (c) temperature, and (d) time. As the aqueous extracts were added to the precursor solution, a color change was observed which indicates a successful reduction process.  Figure 2 indicates the variation in color from light brown to darkish brown which can be attributed to the enhancement in the bioreduction process.

**Figure 3:**
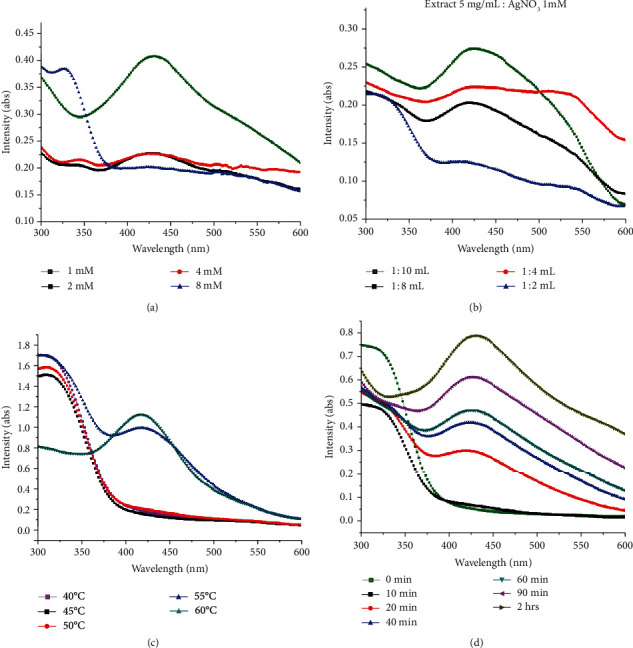
Optimization of different parameters for bioinspired synthesis of silver nanoparticles. UV-vis spectrum of AgNPs mediated by leaf aqueous extracts of *Fagonia indica*: (a) effect of AgNO_3_ concertation, (b) effect of extract and AgNO_3_ (1 mM) ratios on the synthesis of green nanoparticles, (c) effect of temperature, and (d) duration of time for synthesis of silver nanoparticles at different time intervals.

Aqueous extract-mediated reduction of AgNO_3_ in AgNPs was monitored using a spectrophotometer in the UV-visible range. The surface plasmon resonance was found to be ~430 nm. Results of the various optimization parameters are presented in Figures 3(a)–3(d). The concentration of 1 mM AgNO_3_ was found to be effective and yielded silver nanoparticles, while at higher concentrations, the biosynthesis was insignificant as indicated in Figure 3(a). Therefore, the concentration of 1 mM was processed for the further optimization experiments.  Figure 3(b) suggests the variation in concentrations of plant extracts by keeping the concentration of the precursor as 1 mM as indicated in Figure 3(b). Temperature-dependent biosynthesis was performed by applying a varying degree of temperature with a difference of 5°C between the ranges of 40°C to 60°C. It can be observed that below 50°C, there was no formation of AgNPs. Ag nanoparticle formation was indicated at 55°C and 60°C. The temperature of 60°C was considered as the optimum temperature for biomodulated AgNP synthesis. Furthermore, the formation of AgNPs was monitored relative to time. Time-based optimization was carried out from 0 hr to 2 hrs. It was investigated that the biosynthesis was increased relative to the increase in the time period. Biosynthesis was maximum after the duration of 2 hrs as deduced from Figure 3(d). Already optimized parameters were used to carry out the biosynthesis. The results of our study using UV-vis spectroscopy are consistent with the earlier study reported using diverse plant extracts [50–54].

### 3.3. X-Ray Diffraction (XRD)

**Figure 4:**
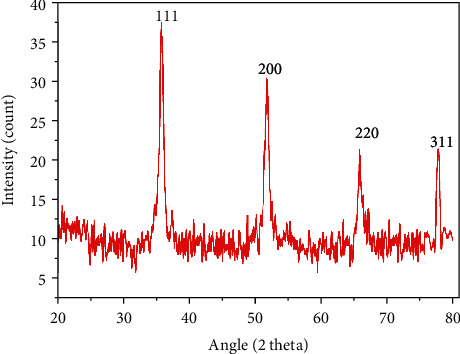
X-ray diffraction (XRD) pattern of green-synthesized AgNPs showing Bragg reflection at angle 2 theta.

XRD analysis was used to examine the crystallinity level of silver nanoparticles.  Figure 4 demonstrates the XRD pattern of biosynthesized AgNPs. The obtained Bragg peaks were found to be consistent with crystallographic reflections from 111 (35.68°), 200 (51.62°), 220 (65.86°), and 311 (77.95°) that corresponds to the JCPDS pattern 04-0783. Average size was calculated as 12.09 nm using the Debye-Scherrer approximation [55]; the results accord with previous results reported by Ullah et al. [56], Prakash et al. [57], and Ajitha et al. [58].

### 3.4. Dynamic Light Scattering (DLS) Analysis

**Figure 5:**
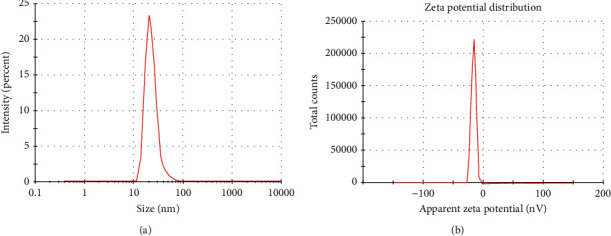
DLS analysis of green-synthesized nanoparticles in (a) zeta size and (b) zeta potential.

The size distribution was further studied using the dynamic light scattering technique. An average hydrodynamic particle size was calculated as 23.68 nm with a polydispersity index near 1. Our result agrees with the particle size of 27 to 32 nm obtained by Kotakadi et al. [59] using the leaf extract of *Catharanthus roseus*. Anandalakshmi and coworkers reported a hydrodynamic size of 150 nm with a diameter of 74 nm [60]. Zeta potential value is an indicator of the stability of the nanoparticle which was calculated as -16.3 mV (Figure 5). The zeta potential is the electric potential resulting from the distribution of charges which indirectly determine the stability of nanoparticles in colloidal suspension [61]. Our results are consistent with the earlier data reported by many research groups [9, 62].

### 3.5. Scanning Electron Microscopy (SEM)

**Figure 6:**
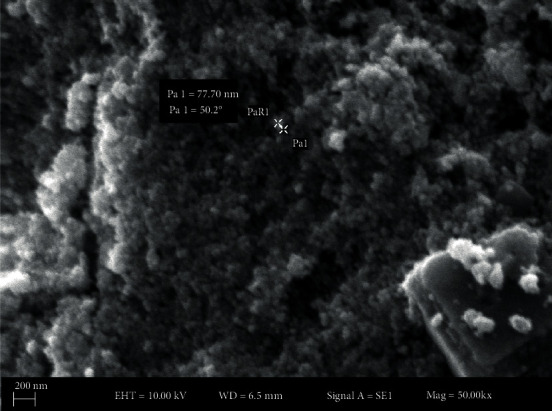
Morphology of AgNPs. SEM micrograph at the scale of 200 nm shows spherical nanoparticles.

SEM micrograph is indicated in Figure 6. The figure shows polydispersed nanoparticles with low agglomeration. The shape of the nanoparticles was observed to be spherical (73.37 at the 200 nm scale). Remya et al. obtained nanoscale silver with a size range of 25-51 nm using *Cassia fistula* flower extract [63]. The same type of results was reported in *Acalypha indica*- and *Syzygium alternifolium*-mediated syntheses of AgNPs [24, 64].

### 3.6. Cytotoxicity

**Figure 7:**
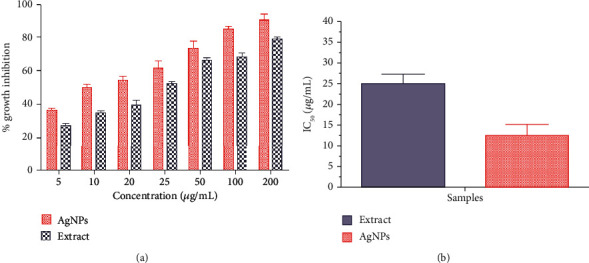
Cytotoxicity of extract and AgNPs in MCF-7 cells. Values are the average ± standard deviation of three experiments conducted in duplicates: (a) percent growth inhibition; (b) IC_50_ concentration.

3-(4,5-Dimethylthiazol-2-Yl)-2,5-diphenyltetrazolium bromide (MTT) cell viability assay was used to determine the cytotoxicity of the extract and AgNPs in MCF-7 cells. The percent growth inhibition of the MCF-7 cells at different doses (5, 10, 20, 25, 50, 100 and 200 *μ*g/mL) was compared to that of untreated cells.  Figure 7(a) shows a concentration-dependent growth inhibition of *in vitro* cultured breast cancer cells. The IC_50_ value was calculated as 12.35 *μ*g/mL for AgNP-treated cells and 25.09 *μ*g/mL for extract-treated cells. This 50% cytotoxic concentration was used for further experiments in this study. Earlier studies report the same type of results studying the effect of green-synthesized AgNPs in MCF-7 cells [22, 65, 66].

### 3.7. Acridine Orange-Ethidium Bromide (AO/EB) Fluorescent Assay

**Figure 8:**
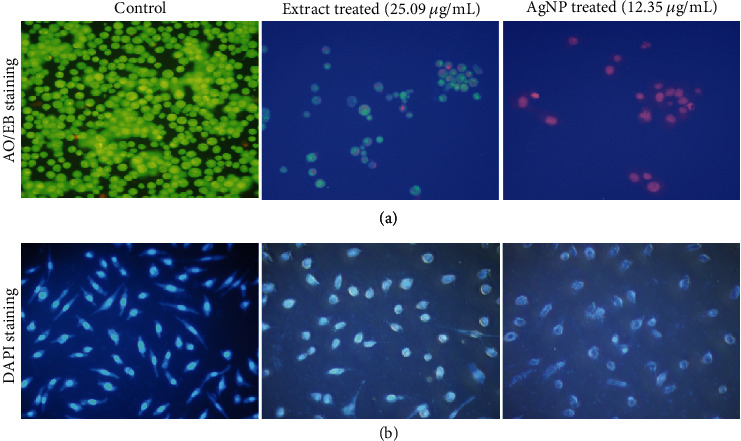
Morphological observation of MCF-7 cells treated with extract and AgNPs. (a) Acridine orange-ethidium bromide (AO/EB) staining. Green indicates viable cells, and reddish/orange staining of the cells indicates apoptotic cells. (b) Morphological changes in the nuclei of MCF-7 cells after treatment with extract and AgNPs induced apoptosis. The changes were observed with DAPI nuclear staining of the treated cells.

The AO/EB fluorescence microscopic staining assay was used to observe the morphological changes in MCF-7 cells. AO/EB staining differentiates between apoptotic and normal cells.  Figure 8 shows the control untreated, extract, and AgNP-treated cells at 12.35 *μ*g/mL (AgNPs) and 25.09 *μ*g/mL (extract) concentrations after 24 hours. The figure shows that the control cells did not change and the cell remains green after staining, whereas the color of the treated cells changed (orange), indicating the apoptotic cells. Moreover, the treated cells show membrane blebbing, shrinkage, and nuclear fragmentation. The same type of membrane changes was observed in MCF-7-treated cells of *Morinda pubescens*-synthesized silver nanoparticles [67], *Teucrium stocksianum* extract-mediated AgNPs [23], *Syzygium aromaticum* extract-mediated AgNPs [22, 68], and *Solanum trilobatum* fruit extract silver nanoparticles [69].

### 3.8. Nuclear Morphology

The outcome of AgNPs on nuclear changes was observed using the DAPI staining assay.  Figure 8 indicates significant changes in the morphology of the chromatin nuclear material after DAPI staining of AgNP and extract-treated cells for 24 hours compared to the untreated control. It can be observed that the control cells have normal rounded nuclei with normal blue color, whereas the treated cells have a bright color, abnormal nuclei, and condensed chromatin with irregular cell structure. These results are consistent with Ciftci et al. [70]. Our results coincide with the previous studies on the influence of green-synthesized AgNPs and plant alkaloids on apoptosis in MCF-7 cells [22, 71, 72]. The apoptosis was further confirmed with Annexin V/PI flow cytometric assay.

### 3.9. Annexin V/Propidium Iodide Apoptosis Detection Assay

**Figure 9:**
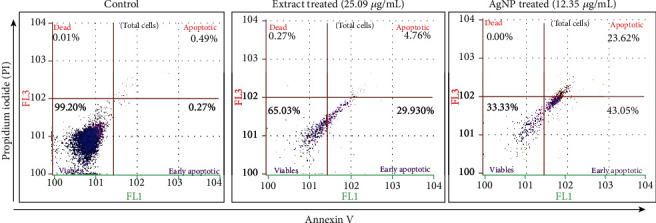
Flow cytometry analysis of MCF-7 cells by double-labelling with Annexin V and PI dyes. The figure shows the early apoptotic, late apoptotic, live, and dead cells given in each quadrant of the untreated growth control cell compared to AgNP-treated cells.

**Figure 10:**
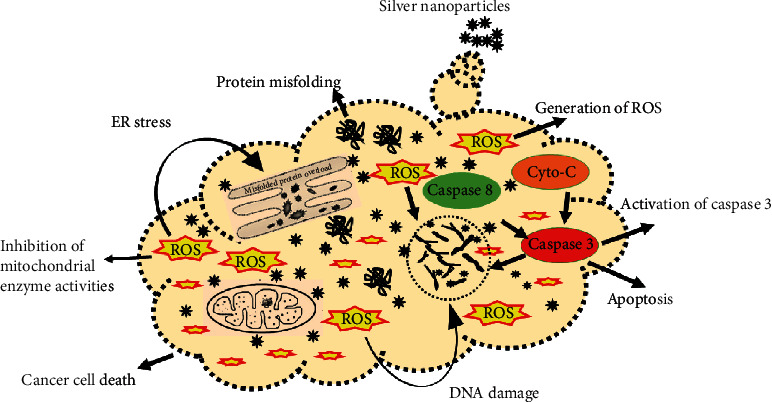
Proposed mechanism of apoptosis induced by caspases and reactive oxygen species.

To further confirm apoptosis, Annexin V/PI staining assay was used. The assay demonstrated the apoptosis in cancer cells exposed to AgNPs (12.35 *μ*g/mL) and extract (25.09 *μ*g/mL) for 24 hours.  Figure 9 shows that untreated cells did not display any significant apoptosis, whereas extract and AgNP-treated cells become apoptotic after 24 hours with early apoptotic cell populations of 43.05% and an apoptotic population of 23.62%. Changes in the population of viable cells indicate that the cell becomes apoptotic due to AgNP-inducing antitumor activities. Similarly, Sriram and colleagues studied the anticancer effects of AgNPs in a tumor model and observed a decrease in the tumor volume [38]. Furthermore, silver nanoparticles induce various biochemical pathways that are involved in the enhanced anticancer activities in MCF-7 cells (Figure 10). Liang et al. [73] and Venugopal et al. [68] observed that green-synthesized silver nanoparticles conjugated with hyaluronic acid-induced apoptosis in cells via autophagy, mitochondrial dysfunction, arrest of the cell cycle, and causing lipid peroxidation.

### 3.10. Caspase 3 and 9 Activities

**Figure 11:**
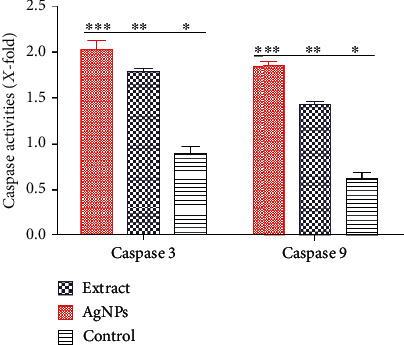
Quantification of caspase 3 and caspase 9 activity in MCF-7 cells exposed to 12.35 *μ*g/mL AgNPs and 25.09 *μ*g/mL extract.

Apoptosis is the course of programmed cellular death that manifests disassembling of the intracellular components while avoiding harm and inflammation of surrounding cells [74]. Caspases are involved in the regulation of inflammatory responses and cell death [75]. Functionally, caspases have two main types, i.e., effector (caspases 3, 6, and 7) and initiator (caspases 2, 8, 9, and 10) caspases [76, 77]. Apoptosis is initiated by the interaction of caspase 3 with caspases 8 and 9. This signal interaction also displays no return in the apoptotic pathway [78]. The apoptosis was further authenticated by measuring the level of the caspase 3 and 9 production in AgNP and extract-treated MCF-7 cells over the untreated control group. Caspases 3 and 9 are the terminal phase inducer of program cell death in cancer cells when activated by external stimuli. The caspase 3 and 9 activities were twofold enhanced in cells exposed to AgNPs and extract compared to control (untreated) cells (Figure 11). The results are consistent with that of Kikuchi et al. [79, 80]. Morphological changes in the membrane and nucleus suggest the possible role of silver nanoparticles in inducing apoptosis in cancer cells [22]. During apoptosis, a series of initiator caspases, e.g., caspase 9, and executioner caspases, e.g., caspase 3, are expressed as an inactive zymogen in the cytoplasm that helps in the program cell death [81, 82]. AgNPs activate these caspases 3 and 9 and some other reactive oxygen species that cause DNA damage, endoplasmic reticulum stress, misfolding of proteins, and apoptosis as shown in Figure 10. It has been reported that on activation, caspase 3 cleaves and translocates caspase-activated DNAse (CAD) that results in DNA fragmentation. DNA fragmentation by endonuclease activity is considered as a prominent event in the apoptosis which occurs in the early stages [83]. The same type of observation was made by Arora et al. studying the effect of AgNPs on cellular responses [84].

### 3.11. Measurement of ROS (Reactive Oxygen Species)

**Figure 12:**
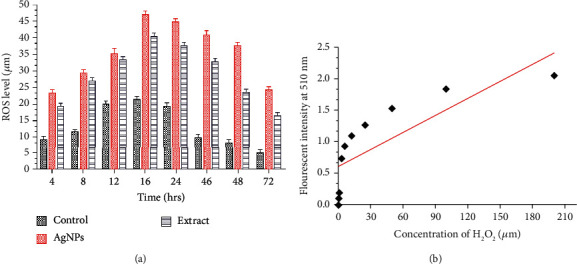
Effects of extract and AgNP exposition on ROS generation in MCF-7 cells. (a) Extent of H_2_O_2_ generation at different time intervals in MCF-7 cells stained with a DCFDH fluorescent probe. (b) Standard curve of H_2_O_2_.

The oxidative pressure made by the free radical produced in response to the external stimuli is the premier cause of apoptosis in cancer cells. Previous research indicates that AgNPs cause oxidative stress and suppress the function of tumor suppressor genes, reduce mitochondrial potential, and induce lipid peroxidation that results in cell apoptosis [85]. A possible mechanism by which apoptosis is manifested by AgNPs is shown in Figure 10. The production of ROS was estimated after treating MCF-7 cells with extract (25.09 *μ*g/mL) and AgNPs (12.35 *μ*g/mL). The estimation of ROS equivalent to H_2_O_2_ (*μ*M) was evaluated compared to the control untreated cell with a different time interval.  Figure 12 demonstrates the quantification of ROS in AgNP- and extract-exposed cells related to the control cells. However, AgNPs were more efficient in the production of ROS as compared to extract-treated cells. This may be due to the effect wherein the plant extract has the ability to scavenge some free radicals. The production of ROS was maximum after 16 hours, and it turned to decrease gradually. The effect of ROS on cellular events depends on the concentrations and duration of treatment. A typical response of cellular events during stress condition is the cell cycle arrest at the G_0_ phase, mitochondrial dysfunction, and apoptosis [86]. The level of ROS-triggering agents is proposed to be used as a therapeutic agent that can selectively kill cancer cells [69, 87]. We observed that the level of ROS generated by AgNPs is on a time-dependent manner. Hsin and colleagues reported that AgNPs generate ROS (reactive oxygen species) in the NIH3T3 cell and induce mitochondria-dependent apoptosis by activating the JNP pathway [88]. ROS are free radicals generated by the biological system for their normal cell functions. The abnormal level of ROS results in the malfunction of cellular components that cause damage to DNA, lipid peroxidation, arresting cell cycle caspase activation, and apoptosis [89].

## 4. Conclusion

In this study, we report a one-step biosynthesis of ecofriendly and stable AgNPs from *Fagonia indica* leaf extract at an optimum condition of 1 mM AgNO_3_ when combined with 5 mg/mL extract in a ratio of 1 : 10 (extract to AgNO_3_) at 60°C for 2 hours. Furthermore, controlled size nanoparticles (10-60 nm) were obtained that were confirmed by XRD, DLS, and SEM analyses. The *Fagonia indica* extract and AgNPs induced anticancer activity in a concentration-dependent manner. The NPs and extract induce membrane permeability, nuclear condensation in an apoptotic manner due to activation of caspases, and generation of reactive oxygen species. Furthermore, these nanoparticles have the potential for the future development of the anticancer drug.

## Data Availability

The data analyzed and mentioned in the text are all included in the manuscript and available to the reader.
